# Response mechanism of *♀ Epinephelus fuscoguttatus* × *♂ Epinephelus lanceolatus* under low-temperature and waterless stresses using TMT proteomic analysis

**DOI:** 10.1007/s00709-021-01654-w

**Published:** 2021-05-05

**Authors:** Xiuping Fan, Qiaoyu Guo, Jiasheng Zhang, Huan Du, Xiaoming Qin

**Affiliations:** 1 College of Food Science and Technology, Guangdong Ocean University, Guangdong Provincial Key Laboratory of Aquatic Product Processing and Safety, Guangdong Provincial Engineering Technology Research Center of Marine Food, Key Laboratory of Advanced Processing of Aquatic Products of Guangdong Higher Education Institution, Zhanjiang, 524088 China; 2 Southern Marine Science and Engineering Guangdong Laboratory, Zhanjiang, 524088 China

**Keywords:** Response mechanism, ♀ *Epinephelus fuscoguttatus*, ♂ *Epinephelus lanceolatus*, Low-temperature, Waterless stresses, TMT proteomic analysis

## Abstract

♀*Epinephelus fuscoguttatu*s × ♂*Epinephelus lanceolatus*, a hybrid grouper created from artificial breeding, has been widely developed over the past decades. However, the study focusing on lukewarm high-protein-content fish species using advanced techniques has rarely been reported. In this work, the TMT (tandem mass tag)-assisted technique was employed to explore its differentially expressed proteins and response mechanisms under low-temperature dormant and waterless stresses. Our findings suggest that 162 and 258 differentially expressed proteins were identified under low-temperature dormant and waterless stresses, respectively. The waterless preservation treatment further identifies 93 differentially expressed proteins. The identified proteins are categorized and found to participate in lipid metabolism, glycometabolism, oxidative stress, immune response, protein and amino acid metabolism, signal transduction, and other functions. Accordingly, the factors that affect the response mechanisms are highlighted to provide new evidences at protein level.

**Supplementary Information:**

The online version contains supplementary material available at 10.1007/s00709-021-01654-w.

## Introduction

Fish with high protein content constitutes important nutrition sources for human health (Golden et al. 2016). With the emerging progress in artificial breeding technology of hybrid grouper, the in-depth evaluation of crude proteins within the products and freshness has drawn extensive attention (Rahimnejad et al. 2015). Benefited from the high water proportion and protease activity as well as the high nutrition, higher-quality products can be expected in the near future by improving the current technology to facilitate the determination of differentially expressed proteins in grouper fillets, and finally, to boom the safe seafood community in a more efficient fashion (Zhang and Xie 2019).

To date, extensive efforts have been devoted towards the low-temperature preservation fishes using ordinary techniques, while the established methods fail to fully reveal the response mechanism of fishes under stress conditions. The response mechanism, in this scene, can be advanced by modern omics techniques (transcriptome, metabolome, proteome, etc.). For instance, transcriptomics in zebra fish and Tilapia mossambica show that the FOXO signal can tailor the lower temperature limit (Hu et al. 2016). Proteomic technology which describes molecular reactions in a more direct way has been widely adopted in mammal hibernation and low-temperature resistance of fishes (Chang et al. 2016; Schrama et al. 2017; Xu et al. 2018). The proteomic study on Brill under temperature and anoxia stresses indicates that both temperature and anoxia gave rise to energy and methionine metabolism, as well as the variation of proteins that were related with fatty acid transportation and amino acid decomposition, among which the key enzymes consisted of ATP synthetase and glucose-6-phosphate dehydrogenase (Pedron et al. 2017). Another study focused on the metabolite indicators of chronic stress of black sea bream (liver proteome) showed that protein-fatty acid-binding protein, heat shock protein, glutamine synthetase, hemoglobin, calmodulin, triosephosphate isomerase, GAPDH, α-enolase, β- tubulin, pyruvate dehydrogenase, and voltage-dependent ionic channel mainly served as biomarkers for chronic stress evaluation of fish (Schrama et al. 2017). A combination of iTRAQ/TMT technique, LC-GC/MS analysis, and parallel reaction monitoring (PRM) can maximally qualify and quantify protein, accompanied with much higher quantitative selectivity and reliability (Chang et al. 2016). The iTRAQ proteomic technique has been adopted to study the low-temperature stress regulation mechanism *of Takifugu fasciatus* (Wen et al. 2019), while its potential use for the lukewarm marine fish, to the best of our knowledge, has rarely been explored.

Bearing these in mind, we studied the metabolism and stress response mechanisms of ♀*Epinephelus fuscoguttatu*s × ♂*Epinephelus lanceolatus* during the low-temperature dormant and waterless preservation processes in this work. The differential proteomic technique was employed to get insightful understanding on the metabolism and stress variations of ♀*Epinephelus fuscoguttatu*s × ♂*Epinephelus lanceolatus* under such stresses, at protein level. The response mechanism is expected to pave a way for further study on the stress mechanism of lukewarm fishes.

## Materials and methods

### Raw materials

*♀ Epinephelus fuscoguttatus* × *♂ Epinephelus lanceolatus* (body length 31.3–34.6 cm, weight 450–600 g) were obtained from the wholescale market for aquatic products in Zhanjiang City, Guangdong Province, China. The aquaculture region locates in the farm on Donghai island. The 1-year-old samples were placed into temporary storage pond (1 × 0.8 m) after receiving from the market, which adopted sand-filtered circulated seawater (salinity 23–25‰) and was oxygenated with air pump, assuring the dissolved oxygen in water with a concentration of 5–8 mg/L. The temporary storage density for fish was determined to be 50 g/L; further experiment was carried out after 48-h temporary storage at 23–25 °C.

*The ♀ Epinephelus fuscoguttatus* × *♂ Epinephelus lanceolatus* after 48-h temporary storage turned into dormant state enabled by a cooling rate of 1–2 °C/h, which was further transferred into the double-packing sterile box filled with oxygen; the humidity was maintained above 9%. The package was put into cold storage (15 °C) to keep the samples alive in waterless condition. The samples were classified into fresh control group (A), dormant group (B), and waterless preservation group (C). Each group consisted of six fish; the experiment was repeated for one time. After that, the fish body was quickly extracted from the box and put into MS-222 anesthesia liquid with a concentration of 300 mg/L. Dissection was carried out after anesthesia, upon which the liver was subjected to quick freezing using liquid nitrogen and then stored in the ultralow temperature refrigerator (− 80 °C) for further use.

### Reagents and instruments

BCA colorimetric was purchased from Sangon Biotech Co., Ltd (Shanghai, China). Tris, NH_4_HCO_3_, and trifluoroacetic acid (TFA) were obtained from Sigma Co., Ltd (USA). SDS, urea, dithiothreitol (DTT), and iodoacetamide (IAA) were bought from Bio-Rad Co., Ltd. Pancreatin was purchased from Promega Co., Ltd. Formic acid (FA) was obtained from Fluka Co., Ltd. Acetonitrile (CAN) was bought from Merke Co., Ltd. TMT 10 plex Isobaric Label Reagent and Pierce™ high pH reverse-phase peptide separation kit was obtained from Thermo Fisher Co., Ltd (USA); SDT lysate consists of 4% SDS, 100 mM Tris–HCl, and 1 mM DTT (pH = 7.6); UA comprises 8 M urea, 150 mM Tris–HCl (pH = 8.0); HPLC mobile phase A, 0.1% FA, mobile phase B, 0.1% FA, and 84% CAN. 5X loading buffer consists of 10% SDS, 0.5% bromophenol blue, 50% glycerinum, 500 mM DTT, and 250 mM Tris–HCl (pH = 6.8).

Easy nLC chromatographic system, Thermo Scientific Q Exactive Mass Spectrometer, microultraviolet spectrophotometer, and Multiskan FC ELIASA were purchased from Thermo Fisher Co., Ltd (USA). The 5430R low-temperature high-speed centrifuge was obtained from Eppendorf Co., Ltd (Germany). EPS601 electrophoresis system was bought from GE Healthcare Co., Ltd (USA). Vacuum centrifugal concentrator was obtained from Eppendorf Concentrator Plus Co., Ltd (Germany). MP Fastprep-24 homogenizer was obtained from MP Biomedicals Co., Ltd (USA). JY92-II ultrasonic cell disruptor was purchased from Ningbo Scientz Biotechnology Co., Ltd (China). Votex QT-1 oscillator was purchased from Shanghai Qite Co., Ltd (China). GNP-9080 constant temperature incubator was obtained from Shanghai Jinghong Co., Ltd (China). LC column–Human 14/Mouse 3 was obtained from Agilent Co., Ltd (USA). Acclaim PepMap100 C18 liquid-phase column was bought from Thermo Scientific Co., Ltd, (100 μm*2 cm, nanoViper C18, 3 μm, 100 Å), USA. EASY C18 analysis column was purchased from Thermo Scientific Co., Ltd (10 cm, ID75 μm, 3 μm, C18-A2), USA.

The analysis software Proteome Discoverer 1.4 (Thermo Scientific Co., Ltd., USA), MASCOT 2.2 (Matrix ScienceCo., Ltd., USA), and Perseus 1.3 (Max Planck Institute for Biochemistry, Martinsried, Germany) were utilized in this work.

### Differential proteomic experiments

Figure S1 gives the schematic diagram for biological analysis, which mainly includes protein extraction, peptide fragment enzymolysis, TMT labeling, chromatographic fractionation, LC–MS/MS data collection, protein identification and quantitation, differential protein screening, clustering analysis of proteins with differential expression, functional annotation, and pathway analysis.

#### Liver protein preparation and electrophoresis identification

The mixed liver of *♀ Epinephelus fuscoguttatus* × *♂ Epinephelus lanceolatus* (0.1 g) was dissolved in SDT lysate and then transferred into a 2-mL centrifuge tube (added with a ¼ inch ceramic bead), which was further homogenized in MP homogenizer (24 × 2) for 60 s (speed 6 M/s) and repeated 2 times. Subsequently, the sample was disrupted in 80 W ultrasonic cell disruptor for 10 times (pulse on 10 s, pulse off 15 s), and then placed in boiling water bath for 15 min. The supernatant was collected after centrifuging at 14,000* g* for 40 min and further filtered using porous membrane (pore size 0.22 μm). The filtrate was collected to determine the amount of protein by BCA method. The protein concentration of each sample was adjusted to 20 μg/mL and added with 5X loading buffer, then placed into boiling water bath for 5 min, followed by electrophoresis using 12.5% SDS-PAGE (constant current 14 mA, 90 min). Coomassie brilliant blue was used for staining. The samples were packaged separately and stored at − 80 °C for further analysis.

#### FASP enzymolysis

The samples (30 μL each) were added with DTT to afford a final concentration of 100 mM, then transferred into boiling water bath for 5 min, followed by cooling at room temperature. Subsequently, 200-μL UA buffer was added to get a homogeneous mixture, which was transferred into a 10-kD ultrafiltration centrifuge and centrifuged at 14,000* g* for 15 min; the filtrate was then discarded (this process was repeated one time). Then, 100-μL IAA (100 mM IAA in UA) buffer was added; the mixture was oscillated at 600 rpm for 1 min and reacted at room temperature for 30 min in dark, followed by centrifuging at 14,000* g* for 15 min. Additional 100 μL UA buffer was again added and centrifuged at 14,000 g for 15 min (repeated two times). Subsequently, 100 μL 100 mM TEAB buffer was added, followed by centrifuging at 14,000* g* for 15 min (repeated two times). Pancreatin decomposition liquor (40 uL, 4 μg Trypsin dissolved in 40 μL 100 mM TEAB buffer) was added and oscillated at 600 rpm for 1 min; the resulting sample was stored at 37 °C for 16–18 h. The mixture was then centrifuged at 14,000* g* for another 15 min, followed by adding with 40 μL 100 mM TEAB buffer (with tenfold dilution) and centrifuging at 14,000* g* for 15 min. Then, the filtrate was collected to quantify the peptides using OD280 method.

#### TMT labeling

TMT analysis was carried out according to the TMT kit using randomly selected 3 samples in each group. Specifically, each aliquot (100 μg peptide equivalent) was reacted with one tube TMT reagent. Sampling labeling is listed in Table S1.

#### RP classification and HPLC separation

The labeled peptide fragments were mixed with equal quantity. The samples after freeze-drying (100 μg) were diluted with 300-μL 0.1 TFA and then transferred into high PH RP spin column and centrifuged to collect the components, which was then added with 300-μL pure water and centrifuged to collect the eluted components and realize gradient elution. The eluted sample was collected and freeze dried, re-dissolved in 12 μL 0.1% FA buffer. The concentration of peptide fragment was determined using OD280.

The samples were separated using Easy nLC liquid system. The buffer consisted of A and B (A, 0.1% FA-water solution; B, 0.1% FA-84% acetonitrile solution) solutions. The chromatographic column was balanced with 95% A solution under a flow speed of 300 nL/min. The gradient of eluent was as follows: 0–50 min (B solution, 0–35%); 50–55 min (B solution, 35–100%); 55–60 min (B solution, 100%).

#### Q-Extractive mass spectrum identification

The as-separated samples were analyzed using Q-Extractive mass spectrum (time 60 min, positive ion detection, 300–1800 m/z parent ion scan). The resolution of 200 m/z first-order mass spectrum was 70,000. The m/z ratios of peptides and polypeptide fragments were collected as follows: ten fragment maps after each full scanning were collected; HCN excitation was utilized with a resolution of 2 m/z. The resolution of 200 m/z second-order mass spectrum was 35,000 (TMT 10 plex).

#### Protein identification and quantitative analysis

The raw data for mass spectrum analysis was RAW format, which were identified and quantitatively analyzed using Mascot2.2 and Proteome Discoverer1.4 software. The protein ratios were calculated as the median of unique peptides of the protein. All peptide ratios were normalized by the median protein ratio, which should be 1 after the normalization.

#### Bioinformatics analysis

Blast2GO was utilized for Gene Ontology (GO) annotation; the general procedures can be summarized as Blast, GO Item Mapping, GO Annotation, and InterProScan (Annotation Augmentation). The KEGG Automatic Annotation Server (KAAS) software was employed for Kyoto Encyclopedia of Genes and Genomes (KEGG) pathway annotation. The Fisher’s exact test was adopted to compare the distribution status of each GO classification or KEGG pathway in protein sets, upon which the enrichment analysis of GO annotation and KEGG pathway annotation in target protein can be realized. The quantitative information of target protein set was firstly subjected to normalization (normalized to (− 1,1) interval). Then, ComplexHeatmap R (R Version 3.4) package was employed to classify the expression quantity of the sample and protein in two dimensionalities (distance algorithm, Euclid; connection mode, average linkage); hierarchical clustering heat map was then generated. The direct and indirect interactions among the target proteins were detected using the information in the databases IntAct (http://www.ebi.ac.uk/intact/main.xhtml) or STRING (http://string-db.org/); the CytoScape (version: 3.2.1) software was used to generate and analyze the interacted network.

#### Statistical analysis

The SPSS software (version 19.0) was used to carry out the statistical analysis in this work, in which the standard *p* < 0.05 was generally regarded as statistically significant. For each experiment, random selection of prepared samples was conducted for three times to guarantee reliable results.

## Results

### Identification of differentially expressed proteins

**Fig. 1:**
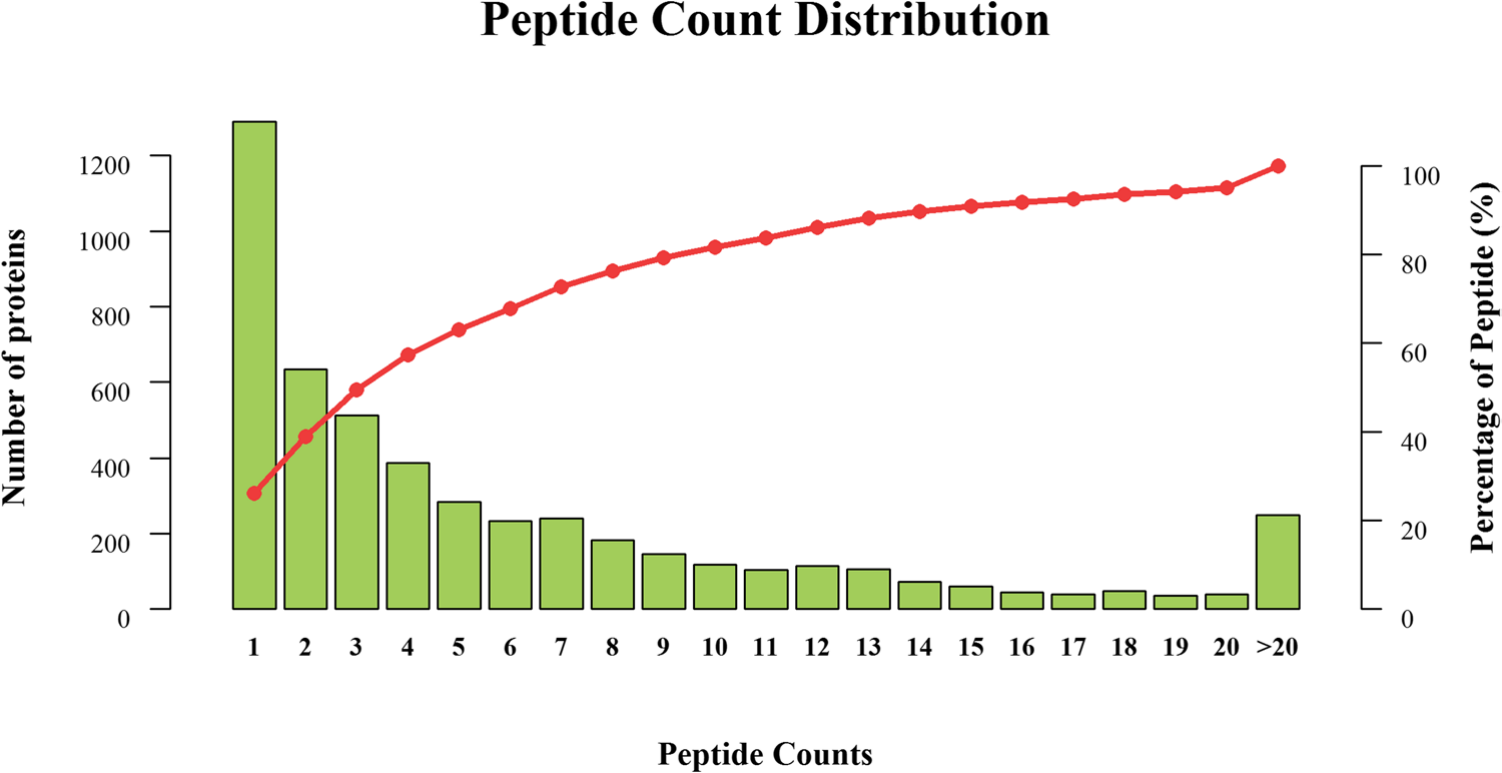
Distribution of peptide fragment number via protein identification

In this work, we found that the numbers of proteins were putative matched 28,777 peptide fragments and 4933 proteins. About 35.09% protein (1731) can identify one characteristic peptide fragment. The proteins that can identify two and three peptide fragments accounted for 15.37% (758) and 10.50% (518), respectively. About 34.66% protein can identify more than four characteristic peptide fragments (Fig. 1).

**Fig. 2:**
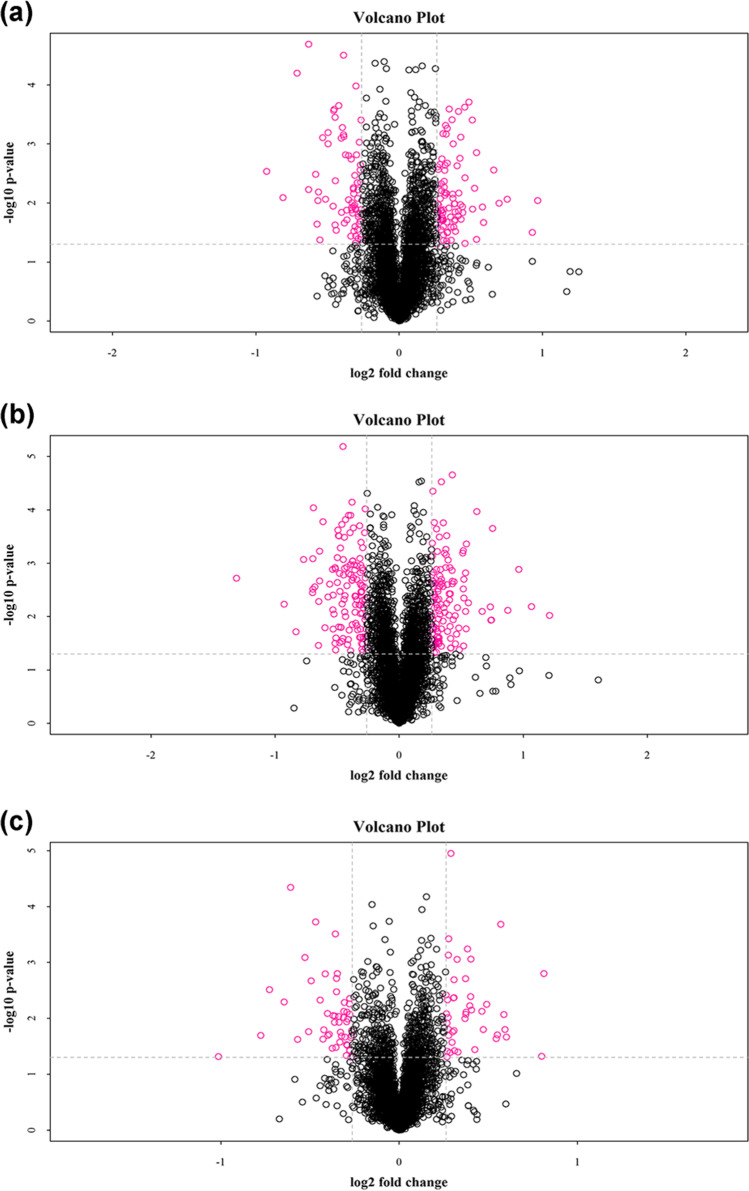
Volcano plots of compared groups. **a** B/A. **b** C/A. **c** C/B. The abscissa is multiples of difference (logarithmic transformation of 2 at the bottom) and the ordinate is the significant difference (*p* value, logarithmic transformation of 10 at the bottom). The red dots denote the differentially significant expressed proteins

The fold-change higher than 1.2 (> 1.2 for upregulation and < 0.83 for downregulation) and *p* < 0.05 constitute the standard for identifying differently expressed proteins. The number of differently expressed proteins in each group is listed in Table S2; the quantification results are presented as volcano plot in Fig. 2. Compared with group A, it is found that a total of 162 and 258 proteins show significant changes in groups B and C, respectively. This indicates that both dormant and waterless preservation processes exert significant impact on protein expression in *♀ Epinephelus fuscoguttatus* × *♂ Epinephelus lanceolatus*, which becomes more evident after treating the samples in 15 °C cold storage to maintain the waterless preservation state, as evidenced by the comparison between groups B and C.

**Fig. 3:**
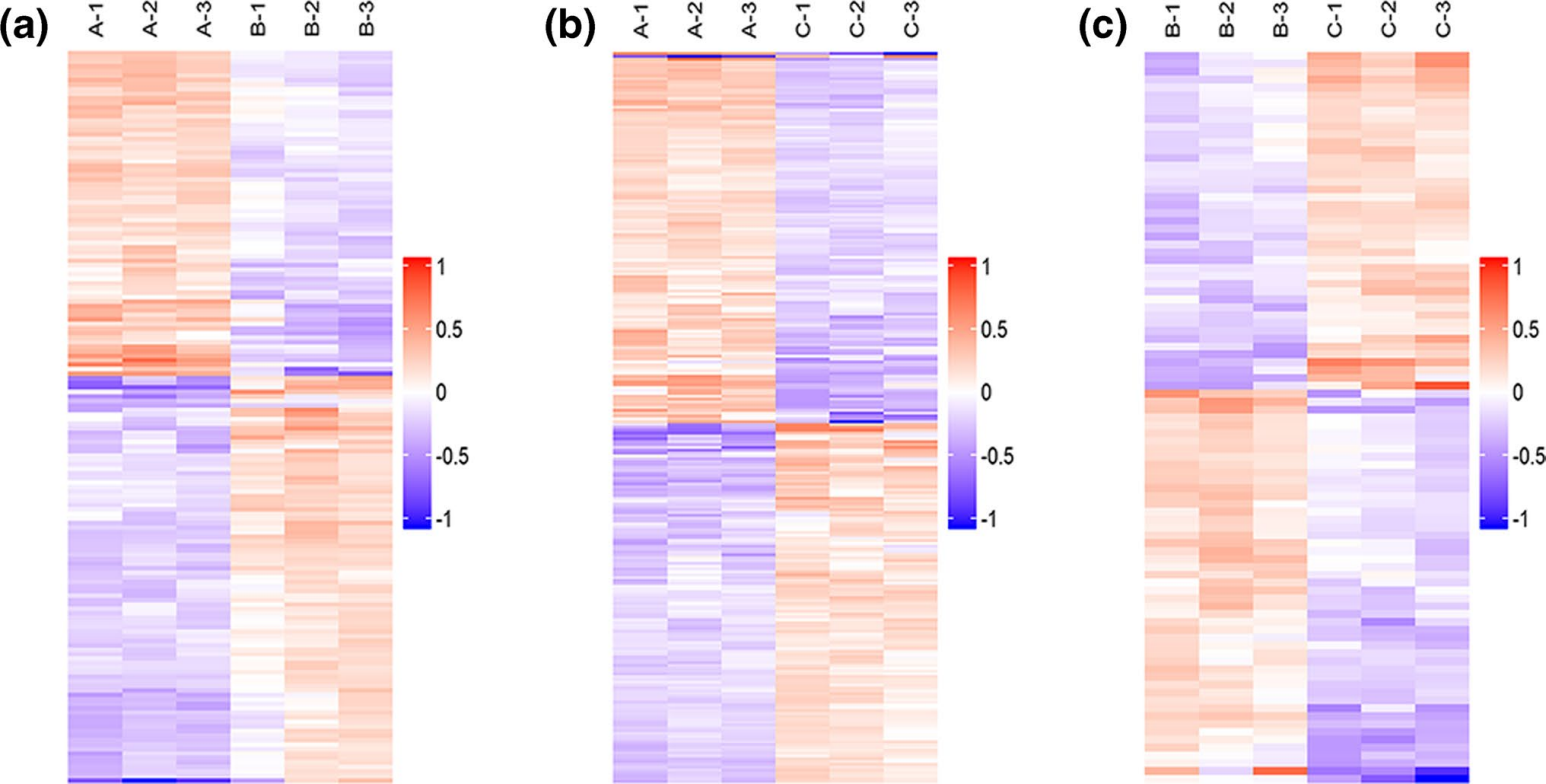
Clustering results of differentially expressed proteins. (a) B/A, (b) C/A, (c) C/B. Red and purple colors denote the significantly upregulated and downregulated proteins, respectively

Clustering analysis was carried out using hierarchical cluster algorithm and presented as heatmap in Fig. 3. Two dimensions (sample and variable) were classified by the algorithm; the results can examine the rationality of screened target proteins, that is, help to determine whether the variation of the expression amount of these target proteins can well represent the significant effect of biological treatment. The clustering results of target proteins can distinguish the protein subsets with different expression modes. Proteins with similar expression modes may share similar function or participate in identical biological ways, or locate in near regulation positions within the pathway.

### Gene Ontology enrichment analysis of differentially expressed proteins

**Fig. 4:**
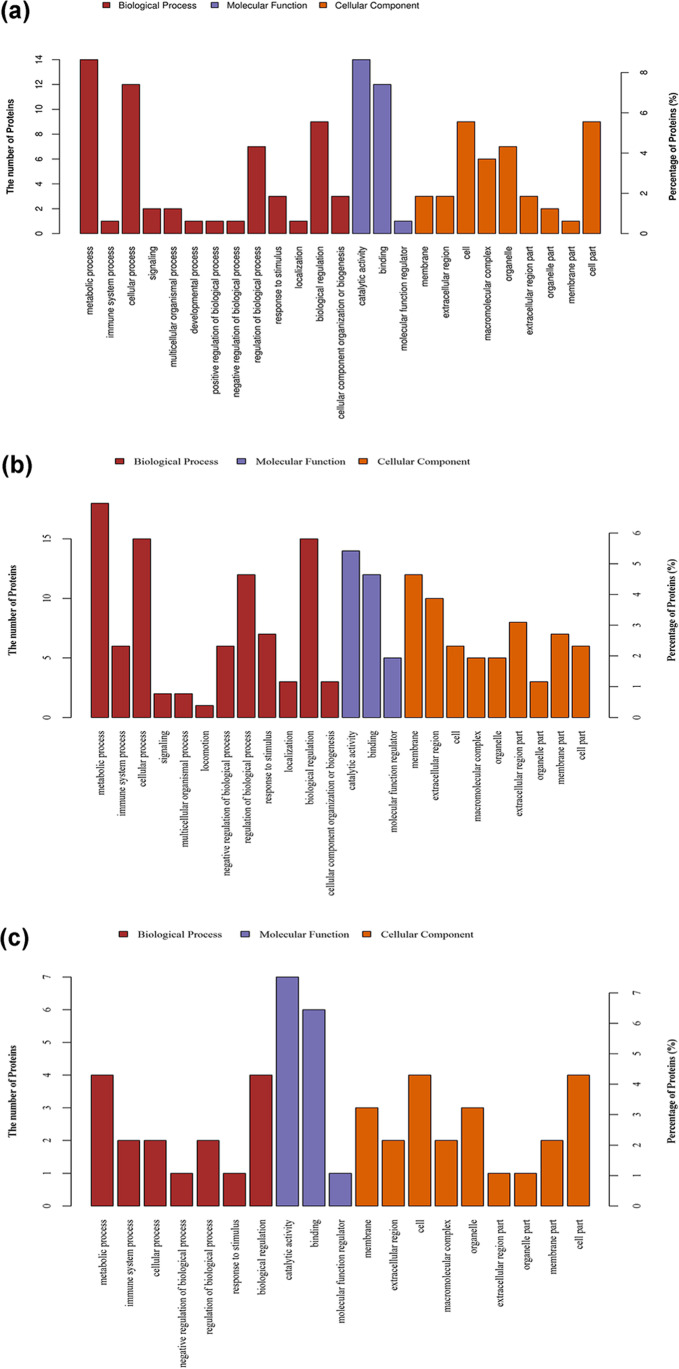
GO enrichment analysis of differentially expressed proteins. **a** B/A. **b** C/A. **c** C/B

GO, a standardized function classification system, provides a set of standard vocabulary that changes dynamically and describes the attributes of gene and genetic products from three aspects: biological process (BP), molecular function (MF), and cellular component (CC). The second-order GO enrichment analysis of 162 low-temperature triggered differentially expressed proteins (group B, Fig. 4a) of *♀ Epinephelus fuscoguttatus* × *♂ Epinephelus lanceolatus* was carried out; the results can be described as follows. These proteins mainly participated in 13 kinds of BP, among which 14 proteins participated in metabolic process (accounted for 8.64%), 12 in cellular process (7.41%), 9 in biological regulation (5.56%), and 7 in regulation of biological function (4.32%). In MF, 14 and 12 proteins individually participated in catalytic activity and binding annotation, accounting for 8.64% and 7.41%, respectively. In CC, the number of proteins that participated in cellular ingredients and annotations was both 9, accounted for 5.56%.

The 258 differentially expressed proteins of *♀ Epinephelus fuscoguttatus* × *♂ Epinephelus lanceolatus* (group C, Fig. 4b) mainly participated in 12 kinds of BP, among which 18 proteins participated in metabolic process (6.98%), 15 in cellular process (5.81%), 15 in biological regulation (5.81%), 12 in regulation of biological function (4.65%), 7 in the response-to-stimulus process (2.71%), 6 in immune system process (2.33%), 6 in negative regulation of biological process (2.33%), 3 in localization, 3 in cellular component organization/biogenesis (1.16%), 2 in signaling and multicellular organismal process (0.78%), and 1 in cellular locomotion (0.39%). Under waterless preservation stress condition, the proteins of *♀ Epinephelus fuscoguttatus* × *♂ Epinephelus lanceolatus* changed significantly in terms of participating in metabolism, biological regulation, stress reaction, immune system regulation, localization, cellular synthesis, and signal transmission process. In MF, 14 and 12 proteins individually participated in catalytic activity and binding annotation, accounting for 5.43% and 4.65%, respectively; 5 proteins participated in molecular function regulator (1.94%). In CC, larger amounts of proteins associated with membrane protein and extracellular region were 12 (4.65%) and 10 (3.88%), respectively; 8 in extracellular region (3.10%), 7 in membrane part (2.71%), 6 in both cell and cell part (2.33%), 5 in both macromolecular complex and organelle (1.94%), and 3 in organelle part (1.16%). The proteins related to the waterless preservation process mainly comprised cell membrane, extracellular region and cell.

Compared with the group B, the 93 differentially expressed proteins in group C mainly participated in seven kinds of BP (Fig. 4c), among which 4 proteins participated in metabolic process (4.30%), 4 in biological regulation (4.30%), 2 in both cellular process and regulation of biological process (2.15%), and 1 in both response to stimulus and negative regulation of biological process (1.08%). In MF, 7 and 6 proteins individually participated into catalytic activity and binding annotation, accounting for 7.53% and 6.45%, respectively; 1 in molecular function regulator (1.08%). In CC, 4 proteins participated in both cell and cell part (4.30%), 3 in both membrane protein and organelle (3.23%), 2 in both extracellular region and macromolecular complex as well as membrane part (2.15%), and 1 in both extracellular region and organelle part (1.08%).

**Fig. 5:**
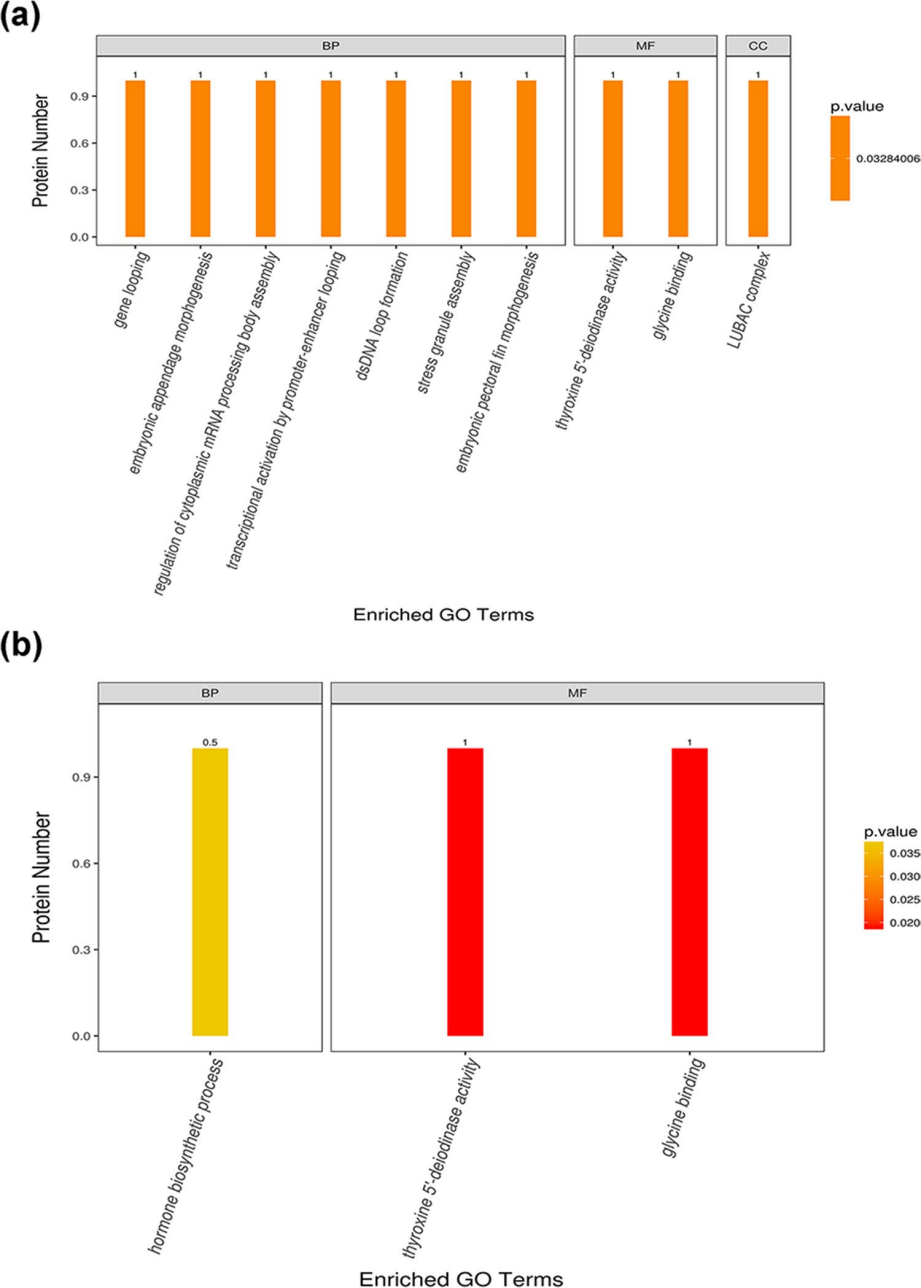
GO enrichment analysis. **a** B/A. **b** C/B

As further displayed in Fig. 5(a), some important BP, including dsDNA loop formation, regulation of cytoplasmic mRNA processing body assembly, gene looping, stress granule assembly, and embryonic pectoral fin morphogenesis, changed significantly in group B when compared with that of group A: accompanied with MF (thyroxine 5′-deiodinase activity, glycine binding, etc.) and positioning protein (LUBAC complex). However, no enrichment was achieved between groups C/A, indicating that the waterless preservation process may disable the important biological processes. A comparison between groups B and C shown in Fig. 5(b) indicates only hormone biosynthetic process (BP); thyroxine 5′-deiodinase activity and glycine binding (MF) changed significantly when the sample was subjected to waterless preservation process after dormant treatment.

### KEGG pathway analysis of differentially expressed proteins

**Fig. 6:**
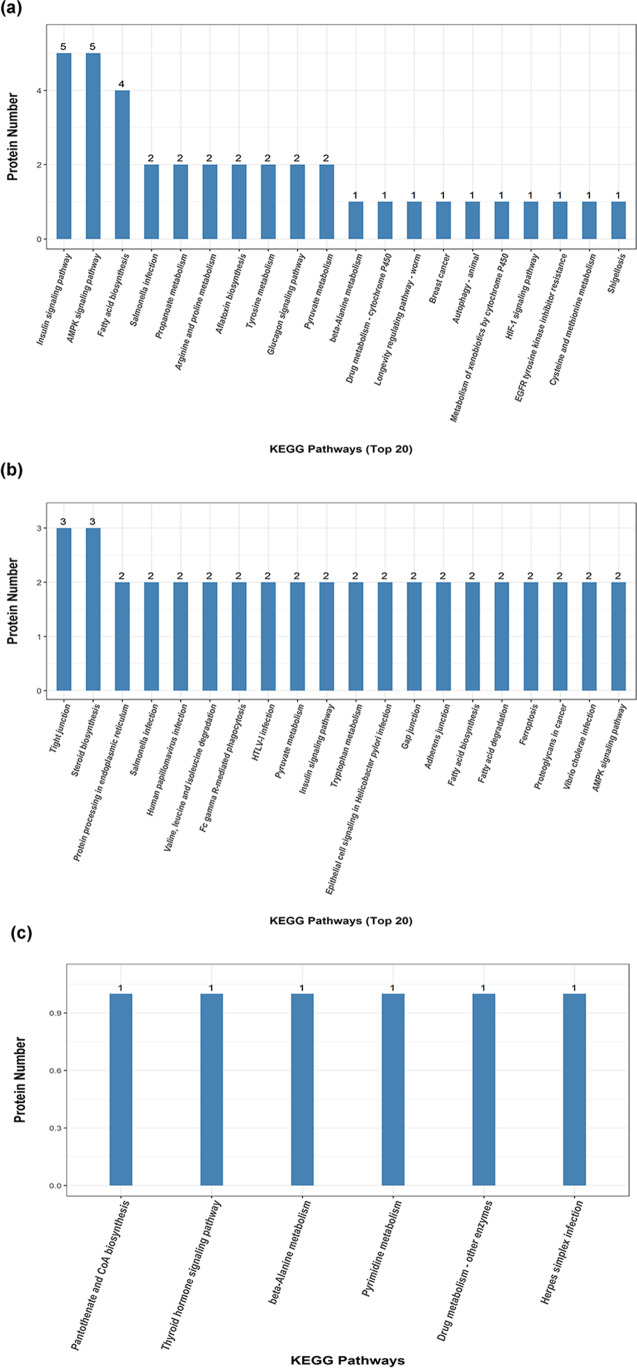
KEGG pathway analysis. **a** B/A. **b** C/A. **c** C/B

In organisms, proteins work coordinately to complete a series of biochemical reaction to share their biological functions. In this regard, pathway analysis serves as a direct and necessary method to systematically get insight into the biological processes and cell status, as well as the pathogenesis and drug action mechanism. KEGG pathway analysis of differentially expressed proteins in the three groups was carried out using Fisher’s exact test; the results are shown in Fig. 6. Compared with group A, 89 differentially expressed proteins in group B participated in 72 metabolic pathways, mainly including insulin signaling pathway, AMPK signaling pathway, and fatty acid biosynthesis. All these proteins show upregulation character. It is noteworthy that fatty acid biosynthesis, AMPK signaling pathway, insulin signaling pathway, and aflatoxin biosynthesis changed significantly (*p* < 0.05); the corresponding proteins all exhibit upregulation character.

In addition, the KEGG signal pathway analysis in group C shows that 120 proteins participated in 99 metabolic pathways, which mainly includes steroid biosynthesis, protein processing in endoplasmic reticulum, and pyruvate metabolism. Typically, the proteins that participated in steroid biosynthesis, fatty acid biosynthesis, phenylalanine, tyrosine, and tryptophan biosynthesis were all upregulated ones, while those that participated in pyruvate metabolism, fatty acid degradation AMPK signaling pathway, valine, leucine and isoleucine degradation, tryptophan metabolism, glucagon signaling pathway, HIF-1 signaling pathway, and glycolysis/gluconeogenesis were all downregulated ones. The activation of AMPK is primarily associated with cellular apoptosis, while the glycolysis can constitute the main metabolic pathway after waterless preservation process.

Taking groups B and C into comparison, it is found that 6 differentially expressed proteins in group C participated into pantothenate and CoA biosynthesis, thyroid hormone signaling pathway, beta-alanine metabolism, drug metabolism, and pyrimidine metabolism. All these proteins were upregulated ones.

Based on the molecular function and metabolic pathways, differentially expressed proteins are mainly classified into four categories. Fifteen proteins associated with fat metabolism mainly participate in the synthesis, transfer, and decomposition of fatty acids, as well as the synthesis and decomposition of phospholipids (Table S3). Sixteen differentially expressed proteins are related to glycometabolism, which mainly participate in gluconeogenesis, transfer, glycolysis, TCA, and oxidative phosphorylation (Table S4). Eight are correlated with oxidative stress (Table S5), twelve are associated with immune response (Table S6), nine in amino acid metabolism, and twelve in signal transduction and other functions (Table S7).

### Q-PCR verification

Table 1 shows the effects of low-temperature and waterless preservation processes on the expression of genes related to lipid metabolism. Compared with that of control group, the relative expression amount of AMPK mRNA in the liver of *♀ Epinephelus fuscoguttatus* × *♂ Epinephelus lanceolatus* shows no significant change in groups B and C. In contrast, the relative expression amounts of RPS6KB mRNA in both groups B and C got significantly elevated, which then returned to the similar level in control group. The significant increment of relative expression amounts of both FAS and ACC genes are also detected in group B (*p* < 0.05), keeping identical tendency with that of enzymatic activity. Compared with that of control group, the relative expression amounts of ACC and FRAP genes in the liver of *♀ Epinephelus fuscoguttatus* × *♂ Epinephelus lanceolatus* also get significantly increased (*p* < 0.05). In addition, there is no identifiable difference of relative expression amount in FAS, ACC, and FABP within the recovery group (*p* > 0.05).

**Table 1:** Effect of low-temperature and waterless preservation on the expression of genes related to lipid metabolism in the liver of *♀ Epinephelus fuscoguttatus* × *♂ Epinephelus lanceolatus*

	AMPK	RPS6KB	FAS	ACC	FABP
Control group	1.20 ± 0.45 ^a^	1.11 ± 0.35 ^b^	0.44 ± 0.11^bc^	0.93 ± 0.44 ^b^	0.72 ± 0.26 ^b^
Dormant group	1.47 ± 0.35 ^a^	1.79 ± 0.48 ^a^	1.09 ± 0.39 ^a^	1.62 ± 0.27 ^a^	0.64 ± 0.19 ^b^
Waterless preservation group	1.46 ± 0.44 ^a^	1.83 ± 0.19 ^a^	0.72 ± 0.20 ^b^	1.78 ± 0.57 ^a^	1.12 ± 0.42 ^a^
Recovery group	1.69 ± 0.66 ^a^	1.17 ± 0.49 ^b^	0.34 ± 0.11 ^c^	1.47 ± 0.61^ab^	0.39 ± 0.29 ^b^
F value	1.518	8.552	18.803	5.149	8.962
*p* value	0.229	0.000	0.000	0.005	0.000

The values denote mean standard deviation of six replicate fishes. The values in the same column sharing different superscripts denote significant difference with *p* value smaller than 0.05, based on Tukey’s test

*AMPK*, adenosine monophosphate activated protein kinase; *RPS6KB*, ribosomal protein S6 kinase β; *FAS*, fatty acid synthetase; *ACC*, acetyl-CoA carboxylase; *FABP*, fatty acid-binding protein

## Discussion

### Response mechanism of lipid metabolism under low-temperature and waterless stress conditions

It is well acknowledged that the mobility of cytomembrane in fish body changes significantly under low-temperature stress (Plewes et al. 2017). In this case, the fatty acids that constitute cytomembrane phospholipid maintain cytomembrane mobility, metabolic enzyme activity, and normal functions of cells through desaturation action, which is one of important mechanisms for fish body to accommodate the low-temperature stress. In this work, FAS (fatty acid synthase) (Menendez and Lupu 2007) and ACC (Acetyl-CoA carboxylase) (Abu-Elheiga et al. 2001) participate in the synthesis and metabolism of fatty acids in the liver of *♀ Epinephelus fuscoguttatus* × *♂ Epinephelus lanceolatus*, showing upregulation tendency under low-temperature conditions (compared with group A), suggesting strengthened synthesis of fatty acids in the liver, decreased energy consumption, and metabolism. Compared with group B, however, the samples in group C show upregulation of FAS and ACC, signifying the weakened synthesis of fatty acids in the liver, due to the stress from exposing in air atmosphere.

FABP (fatty acid-binding protein) (Lei et al. 2020) that participates in the transfer process of fatty acids was upregulated due to strengthened synthesis of fatty acids under low temperature, while the proteins CPT-1 (carnitine O-palmitoyltransferase 1) (Coccia et al. 2014) and ACAC (acetyl-coa acyltransferase) (Ayisi et al. 2018) related with the oxidative decomposition of fatty acids were downregulated in groups B and C. This finding suggests that the energy supply for the oxidation of very-long-chain fatty acids was suppressed. In groups B and C, PLB-1 (phospholipase B-like 1) (Li et al. 2018) that participated in the synthesis of phospholipid got upregulated, indicating relieved decomposition of cytomembrane phospholipid. As such, the as-synthesized fatty acids may participate in phospholipid synthesis to resist the potential change of cytomembrane mobility under low-temperature dormant and waterless preservation status. In contrast, CPT-1 (carnitine O-palmitoyltransferase 1) (Yu et al. 2020) participating in the oxidation of fatty acids was downregulated. The combined results clearly demonstrate that the low-temperature waterless stress changes the fat components of cytomembrane, upon which the fish body maintains cytomembrane mobility through phospholipid synthesis.

### Response mechanism of glycometabolism under low-temperature and waterless stress conditions

As for the glycometabolism-related proteins, G6P (glucose-6-phosphatase, G6P) (Guillen et al. 2019) was downregulated in groups B and C, due to the weakened effect of gluconeogenesis that may be derived from the hysteresis of protein expression. PFK (ATP-dependent 6-phosphofructokinase) (Moniruzzaman et al. 2020), ADH (alcohol dehydrogenase) (White et al. 2020), and PDK2 (pyruvate dehydrogenase [lipoamide] kinase isozyme 2) (Wu et al. 2018) were downregulated in group B, which is attributed to the decreased requirement on energy of fish body and suppressed energy supply for glycolysis. However, the downregulation of PDK-2 and upregulation of KHK (ketohexokinase) (Wang et al. 2019) in group C indicate the abnormal metabolism when the fish body was in stress condition, in which ketose participates in the energy supply for glycometabolism through glycolysis. Compared with group B, the upregulation of PFK while downregulation of KHK in group C were identified, suggesting that the fish body used the energy from glycolysis to resist the waterless stress. ATP-CS (ATP-citrate synthase) (Jesus et al. 2018) and ICDHX1 (Isocitrate dehydrogenase X1) (Tang et al. 2018) were downregulated in group B, while GLUT2 (solute carrier family 2 facilitated glucose transporter member 2, Slc2 a 2) (Blanco et al. 2017), ATP-CS, and AACP (ATP carrier protein) were upregulated in group C.

Taking together, these findings signify that the glycometabolism of *♀ Epinephelus fuscoguttatus* × *♂ Epinephelus lanceolatus* was significantly affected under low-temperature dormant and waterless stress, while the proteins differ from mRNA in terms of expression level, probably due to the regulation effect during the protein translation process induced by gene.

### Response mechanism of oxidative stress under low temperature and waterless stress conditions

The upregulation of CIRP (cold inducible RNA binding protein) (Zhu et al. 2016) in group B was indicative of enhanced anti-hypoxia ability for the sample under low-temperature dormant status; the upregulation of GST (alpha-class glutathione S-transferase) (Ozaslan et al. 2017) in both groups B and C signifies the anti-oxidation ability was enhanced. The upregulation of ALDH (aldehyde dehydrogenase (NAD +)) (Xu et al. 2020) in group C indicates that the fish body can resist the damage from low-temperature waterless stress by initiating the anti-oxidation mechanism. The downregulation of GSH (Lunardelli et al. 2018) and HSP70 (Heat shock proteins) (Liu et al. 2017) that are related with oxidative stress and detoxification were also identified under low-temperature stress. The upregulation of ALDH (aldehyde dehydrogenase (NAD +)), CAT, and CP450 were identified when the samples were subjected to low-temperature stress.

### Immune response under low-temperature and waterless stress conditions

The proteins participating in the regulation of immune functions, including immunoglobulin (IgL, IgH), alpha-2-macroglobulin, complement component 4, hemoglobin subunit alpha-1, and ferritin middle subunit, were all upregulated in groups B and C. The downregulation of complement C1q-like protein 3 and complement component C9 indicates that acute low temperature will give rise to damaged immune response, while the upregulation of complement C1q-like protein 3 in group C clearly demonstrates that the sample changed the immune regulation function and initiated new immune response mechanism after low-temperature waterless treatment. BPI (bactericidal/permeability-increasing protein) (Yang et al. 2019) and LPS (lipopolysaccharide) (Jiang et al. 2017) were both upregulated in groups B and C, indicating that low-temperature stress and temperature variation failed to resist the immune response mediated by LPS. In addition, the downregulation of LAAO (L-amino acid oxidase) (Suwannapan et al. 2018) in groups B and C denotes that the immune regulation induced by oxidative stress was affected.

### Regulation of protein and amino acid metabolism under low-temperature and waterless stress conditions

PDH (proline dehydrogenase) and agmatinase are known to participate in the metabolism of arginine and proline; their upregulation in group B signifies the increased decomposition of arginine and proline (Suwannapan et al. 2018; Shao et al. 2018). However, no significant change was identified for the key enzyme P5CS (Δ1-Pyrroline-5-carboxylate synthase) (Li and Wu 2018) that participates in the synthesis of proline. It was found that the proline content decreased in the muscle of *♀ Epinephelus fuscoguttatus* × *♂ Epinephelus lanceolatus* and began to increase at 12 °C (dormant temperature). In contrast, arginine decreased to a minimum level at 12 °C with the increase of decomposition and metabolism. Except LAAO, all other proteins were upregulated under low-temperature dormant stress, signifying the increase of amino acid decomposition and metabolism. The downregulation of LAAO and GluAP is indicative of newly synthesized amino acids or proteins.

### Response mechanism of signal transduction proteins under low-temperature and waterless stress conditions

Protein phosphorylation acts as an important regulation mechanism when the organism cells respond to various stress signals, while protein kinase affects signal transduction of cells by catalyzing the phosphorylation of substrate proteins. During the waterless preservation process, the upregulation of serine-threonine protein kinase Sgk2 (Caballero-Solares et al. 2018) under low-temperature stress indicates that the fish body can trigger the dormant status to resist the potential damage of liver, by virtue of the phosphorylation of functional proteins (such as enzymes, receptors, transport proteins, regulatory proteins, nuclear proteins). Both groups B and C show that RPS6KB (ribosomal protein S6 kinase) (Abasubong et al. 2019) was upregulated, attributable to the fact that RPS6KB/mTOR (mammalian target of rapamycin) signal participated in the energy metabolism and stress response (Dai et al. 2018). In addition, the downregulation of PP1 (protein phosphatase 1) (Richard et al. 2018) in group B is due to the resistance of fish liver against glycogen synthesis. Another proof that shows the significantly changed lipid metabolism under waterless stress can be confirmed by the upregulation of PI3K (phosphatidylinositol 3-kinase) (Song et al. 2018) and cAMP-RP19 (cAMP-regulated phosphoprotein 19-like) (Montero et al. 2016). CBPs (calcium-binding protein) (Thiruketheeswaran et al. 2016) is generally related with glycoprotein folding and protein synthesis, which maintains the protein activity within animal body to guarantee normally stable structure. However, the protein structure was probably affected under low-temperature and waterless stress, as evidenced by the downregulation of CBPs, which deserves further exploration.

### Regulation effect of AMPK signal pathways under low-temperature and waterless stress conditions

Phosphorylation of AMPK can affect the expression of downstream TOR and other target genes, regulating the energy metabolism, amino acid, and fatty acid metabolism. The ATP level can be regulated by AMPK, which can experience the change of AMP level within the cells and further provide energy for the cells during the stress reactions. For the fishes under hypoxia, hungry, and low-temperature stresses, they are capable of regulating the energy metabolism and stress response by AMPK signal pathway. In this work, the proteins (FAS, ACC, RPS6KB) participating in AMPK signal pathway were all upregulated in groups B and C. Identical variation tendency of FAS, ACC, and RPS6KB mRNA were demonstrated in Q-PCR results, along with the upregulation of stress protein GST and enhanced expression of mRNA. The expression of AMPK protein and mRNA show no significant change, indicating that the energy (lipid) metabolism and stress response of *♀ Epinephelus fuscoguttatus* × *♂ Epinephelus lanceolatus* were regulated by AMPK signal pathway, which finally improved the survivability of fish body under low-temperature and waterless stresses.

## Conclusion

In this work, the TMT-assisted strategy was employed to explore the differentially expressed proteins of *Epinephelus fuscoguttatus* × *♂ Epinephelus lanceolatus*. Low-temperature dormant and waterless preservation processes were proved to exert significant effect on the energy metabolism (glucide, lipid, amino acid), oxidative stress, detoxification function of the liver, and immune response of *♀ Epinephelus fuscoguttatus* × *♂ Epinephelus lanceolatus*. A total of 162 and 258 differentially expressed proteins were identified under low-temperature dormant and waterless stresses, respectively. The waterless preservation treatment for 8 h further shows 93 differentially expressed proteins. The main conclusions are as follows:

1. The low-temperature dormant and waterless preservation treatments resulted in the upregulation of FAS, ACC, and FABP that participate in the synthesis and transfer of fatty acids, as well as the downregulation of CPT-1 and ACAC that participate in the oxidative decomposition of fatty acids. This finding suggests that *♀ Epinephelus fuscoguttatus* × *♂ Epinephelus lanceolatus* experienced weakened metabolism, decreased energy consumption, and enhanced synthesis of fatty acids under low-temperature waterless preservation stress. The as-synthesized fatty acids may predominantly participate in the synthesis of phospholipid to resist change of cytomembrane mobility.
2. The proteins CIRP and GST related with the oxidative stress and detoxification were upregulated, while GSH and HSP70 were downregulated for the samples under low-temperature stress. Further waterless stress contributed to upregulation of HSP70 and downregulation of ALDH, CAT, and CP450, suggesting the existence of diverse stress response mechanisms of the fish body.
3. Simple low-temperature stress gave rise to the upregulation of IgL, C4, and Gimap4, while further waterless stress resulted in the upregulation of IgMuH, C1qP3, and BPI/LBP, indicating the different immune response models when *♀ Epinephelus fuscoguttatus* × *♂ Epinephelus lanceolatus* was subjected to low-temperature and waterless stresses.
4. The stress signal RPS6KB, glycometabolism signal proteins PRPS2, and cAMP-RP19 in the liver of *♀ Epinephelus fuscoguttatus* × *♂ Epinephelus lanceolatus* were upregulated under low-temperature dormant and waterless preservation stresses, which participated in the regulation of energy and stress molecules, while Gimap4 participated in the immune response regulation under low temperature.
5. The AMPK signal pathway regulated the lipid metabolism and stress response of *♀ Epinephelus fuscoguttatus* × *♂ Epinephelus lanceolatus* under low-temperature and waterless stresses, thereby advancing its survivability.

This work is expected to reveal the potential changes of differentially expressed changes and the response mechanisms of *♀ Epinephelus fuscoguttatus* × *♂ Epinephelus lanceolatus* during low-temperature treatment.

## Supplementary Information

Below is the link to the electronic supplementary material.

Supplementary file1 (DOCX 195 KB)

## Data Availability

The datasets used or analyzed during the current study are available from the corresponding author on reasonable request.

## References

[CR1] Abasubong KP, Liu WB, Adjoumani YJJ (2019). Xylooligosaccharides benefits the growth, digestive functions and TOR signaling in Megalobramaamblycephala fed diets with fish meal replaced by rice protein concentrate. Aquaculture.

[CR2] Abu-Elheiga L, Matzuk MM, Abo-Hashema AAH (2001). Continuous fatty acid oxidation and reduced fat storage in mice lacking Acetyl-CoA Carboxylase 2. Science.

[CR3] Ayisi CL, Yamei C, Zhao JL (2018). Genes, transcription factors and enzymes involved in lipid metabolism in fin fish. Agri Gene.

[CR4] Blanco AM, Bertucci JI, Ramesh N (2017). Ghrelin facilitates GLUT2-, SGLT1- and SGLT2-mediated intestinal glucose transport in goldfish (*Carassius auratus*). Sci Rep.

[CR5] Caballero-Solares A, Xue X, Parrish CC (2018). Changes in the liver transcriptome of farmed Atlantic salmon (*Salmo salar*) fed experimental diets based on terrestrial alternatives to fish meal and fish oil. BMC Genomics.

[CR6] Chang H, Jiang SF, Dang K (2016). iTRAQ-based proteomic analysis of myofibrillar contents and relevant synthesis and proteolytic proteins in soleus muscle of hibernating Daurian ground squirrels (*Spermophilus dauricus*). Proteome Science.

[CR7] Coccia E, Varricchio E, Vito P (2014). Fatty acid-specific alterations in Leptin, PPARα, and CPT-1 gene expression in the rainbow trout. Lipids.

[CR8] Dai YJ, Jiang GZ, Yuan XY (2018). High-fat-diet-induced inflammation depresses the appetite of blunt snout bream (*Megalobrama amblycephala*) through the transcriptional regulation of leptin/mammalian target of rapamycin. Br J Nutr.

[CR9] Golden CD, Allison EH, Cheung WWL (2016). Nutrition: fall in fish catch threatens human health. Nature.

[CR10] Guillen AG, Borges ME, Herrerias T (2019). Effect of gradual temperature increase on the carbohydrate energy metabolism responses of the Antarctic fish *Notothenia rossii*. Mar Environ Res.

[CR11] Hu P, Liu M, Liu Y (2016). Transcriptome comparison reveals a genetic network regulating the lower temperature limit in fish. Sci Rep.

[CR12] Jesus TF, Rosa IC, Repolho T (2018). Different ecophysiological responses of freshwater fish to warming and acidification. Comp Biochem Physiol A MolIntegr Physiol.

[CR13] Jiang J, Yin L, Li JY (2017). Glutamate attenuates lipopolysaccharide-induced oxidative damage and mRNA expression changes of tight junction and defensin proteins, inflammatory and apoptosis response signaling molecules in the intestine of fish. Fish Shellfish Immunol.

[CR14] Lei C, Li M, Zhang M (2020). Cloning, molecular characterization, and nutritional regulation of fatty acid-binding protein family genes in gold pompanos (*Trachinotus ovatus*). Comp Biochem Physiol B: Biochem Mol Biol.

[CR15] Li P, Wu G (2018). Roles of dietary glycine, proline, and hydroxyproline in collagen synthesis and animal growth. Amino Acids.

[CR16] Li K, Egelandsdal B, Olsen RE (2018). Hydrolysis activity of pyloric cecal enterocytes of brown trout (*Salmo trutta*) toward monoacylglycerol and lysophosphatidylcholine. Lipids.

[CR17] Liu Y, Ma D, Zhao C (2017). The expression pattern of hsp70 plays a critical role in thermal tolerance of marine demersal fish: multilevel responses of *Paralichthys olivaceus* and its hybrids (*P. olivaceus* ♀ × *P. dentatus* ♂) to chronic and acute heat stress. Mar Environ Res.

[CR18] Lunardelli B, Cabral MT, Vieira CED (2018). Chromium accumulation and biomarker responses in the Neotropical fish *Prochilodus lineatus* caged in a river under the influence of tannery activities. Ecotoxicol Environ Saf.

[CR19] Menendez JA, Lupu R (2007). Fatty acid synthase and the lipogenic phenotype in cancer pathogenesis. Nat Rev Cancer.

[CR20] Moniruzzaman M, Kumar S, Das D (2020). Enzymatic, non enzymatic antioxidants and glucose metabolism enzymes response differently against metal stress in muscles of three fish species depending on different feeding niche. Ecotoxicol Environ Saf.

[CR21] Montero J, Gomez-Abellan V, Arizcun M (2016). Prostaglandin E2 promotes M2 polarization of macrophages via a cAMP/CREB signaling pathway and deactivates granulocytes in teleost fish. Fish Shellfish Immunol.

[CR22] Ozaslan MS, Demir Y, Kufrevioglu OI (2017). Some metals inhibit the glutathione S-transferase from Van Lake fish gills. J Biochem Mol Toxicol.

[CR23] Pedron N, Artigaud S, Infante JLZ (2017). Proteomic responses of European flounder to temperature and hypoxia as interacting stressors: differential sensitivities of populations. Sci Total Environ.

[CR24] Plewes MR, Burns PD, Graham PE (2017). Effect of fish meal supplementation on spatial distribution of lipid microdomains and on the lateral mobility of membrane-bound prostaglandin F2α receptors in bovine corpora lutea. Domest Anim Endocrinol.

[CR25] Rahimnejad S, Bang IC, Park JY (2015). Effects of dietary protein and lipid levels on growth performance, feed utilization and body composition of juvenile hybrid grouper, *Epinephelus fuscoguttatus × E. lanceolatus*. Aquaculture.

[CR26] Richard CA, Rincheval V, Lassoued S (2018). RSV hijacks cellular protein phosphatase 1 to regulate M2–1 phosphorylation and viral transcription. PLoS Pathog.

[CR27] Schrama D, Richard N, Silva TS (2017). Enhanced dietary formulation to mitigate winter thermal stress in gilthead sea bream (*Sparus aurata*): a 2D-DIGE plasma proteome study. Fish Physiol Biochem.

[CR28] Shao Y, Zhang S, Li C (2018). Identification of agmatinase from *Vibrio splendidus* and its roles in modulating arginine metabolism of *Apostichopus japonicas*. Aquaculture.

[CR29] Song Y, Liu B, Xu P (2018). Oxidized fish oil injury stress in *Megalobrama amblycephala*: evaluated by growth, intestinal physiology, and transcriptome-based PI3K-Akt/NF-κB/TCR inflammatory signaling. Fish Shellfish Immunol.

[CR30] Suwannapan W, Chumnanpuen P, E-kobon T (2018). Amplification and bioinformatics analysis of conserved FAD-binding region of L-amino acid oxidase (*LAAO*) genes in gastropods compared to other organisms. Comput Struct Biotechnol J.

[CR31] Tang X, Liu F, Sheng X (2018). Recombinant NADP-dependent isocitrate dehydrogenase of *Edwardsiella tarda* induces both Th1 and Th2 type immune responses and evokes protective efficacy against edwardsiellosis. Vaccine.

[CR32] Thiruketheeswaran P, Kiehl E, D’Haese J (2016). Soluble calcium-binding proteins (SCBPs) of the earthworm *Lumbricus terrestris*: molecular characterization and localization by FISH in muscle and neuronal tissue. Histochem Cell Biol.

[CR33] Wang X, Chang L, Zhao T (2019). Metabolic switch in energy metabolism mediates the sublethal effects induced by glyphosate-based herbicide on tadpoles of a farmland frog *Microhyla fissipes*. Ecotoxicol Environ Saf.

[CR34] Wen X, Zhang X, Hu Y (2019). iTRAQ-based quantitative proteomic analysis of *Takifugu fasciatus* liver in response to low-temperature stress. J Proteomics.

[CR35] White LJ, Sutton G, Schechonge A (2020). Adaptation of the carbamoyl-phosphate synthetase enzyme in an extremophile fish. R Soc Open Sci.

[CR36] Wu S, Yu L, Fu X (2018). iTRAQ-based proteomic profile analysis of ISKNV-infected CPB cells with emphasizing on glucose metabolism, apoptosis and autophagy pathways. Fish Shellfish Immunol.

[CR37] Xu DD, You QC, Chi CF (2018). Transcriptional response to low temperature in the yellow drum (*Nibea albiflora*) and identification of genes related to cold stress. Comp Biochem Physiol D Genomics Proteomics.

[CR38] Xu L, Guo W, Liu W (2020). Metabolites analysis for cold-resistant yeast (*Wickerhamomyces anomalus*) strains own antioxidant activity on cold stored fish mince. Food Chem.

[CR39] Yang D, Han Y, Chen L (2019). A bactericidal permeability-increasing protein (BPI) from manila clam *Ruditapes philippinarum*: investigation on the antibacterial activities and antibacterial action mode. Fish Shellfish Immunol.

[CR40] Yu T, Chen YK, Chen XM (2020). The effect of oxidized fish oil on lipid metabolism in *Rhynchocypris lagowski*Dybowski. Aquaculture Reports.

[CR41] Zhang X, Xie J (2019). Analysis of proteins associated with quality deterioration of grouper fillets based on TMT quantitative proteomics during refrigerated storage. Molecules.

[CR42] Zhu X, Buhrer C, Wellmann S (2016). Cold-inducible proteins CIRP and RBM3, a unique couple with activities far beyond the cold. Cell Mol Life Sci.

