# Supplementary material for: Response mechanism of ♀ Epinephelus fuscoguttatus × ♂ Epinephelus lanceolatus under low-temperature and waterless stresses using TMT proteomic analysis
Source: Protoplasma. 2021 May 5;259(1):217–31. doi: 10.1007/s00709-021-01654-w (PMC8752522; doi:10.1007/s00709-021-01654-w)
Supplement: Supplementary file 1 — Supplementary file1 (DOCX 195 KB) [file 709_2021_1654_MOESM1_ESM.docx]

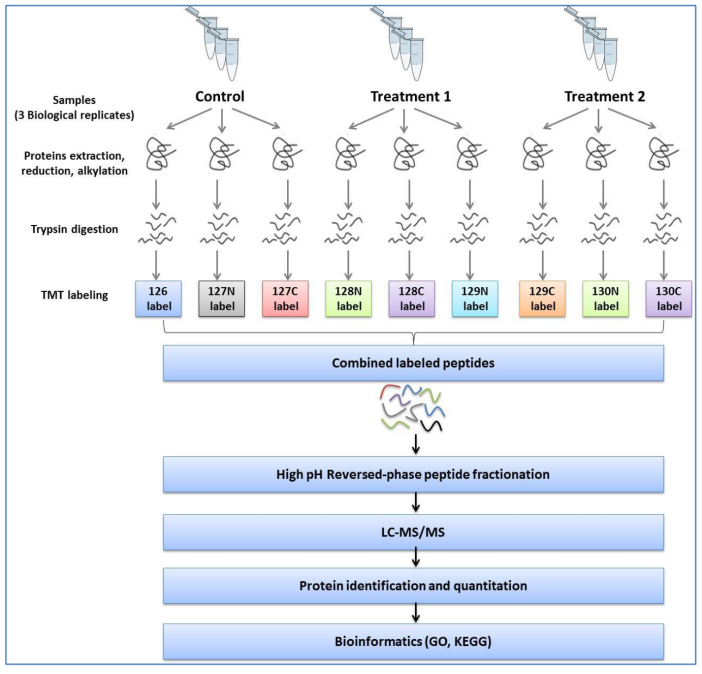


**Fig. S1** Flow chat for biological analysis of*♀Epinephelus fuscoguttatus × ♂Epinephelus lanceolatus*.

**Table S1** Sample name and TMT tag information.

| TMT labeling | 126 | 127N | 127C | 128N | 128C | 129N | 129C | 130N | 130C | 131 |
| --- | --- | --- | --- | --- | --- | --- | --- | --- | --- | --- |
| Sample name | A1 | A2 | A3 | B1 | B2 | B3 | C1 | C2 | C3 | － |

A1, A2 and A3 are three repeated samples in the control group; B1, B2 and B3 are three repeated samples in low-temperature dormant group; C1, C2 and C3 are three repeated samples in low-temperature anhydrous alive group.

**Table S2** Quantification results of differentially expressed proteins.

| Group comparison | Up* | Down* | All* |
| --- | --- | --- | --- |
| B/A | 90 | 72 | 162 |
| C/A | 128 | 130 | 258 |
| C/B | 43 | 50 | 93 |

*: “Up” and “Down” represent the differentially expressed proteins with up-regulation and down-regulation, respectively; “All” represents all differentially expressed proteins.

**Table S3** Variation of Differentially expressed proteins associated with fat metabolism

| Protein IDs | Protein name | Fold change | | | Metabolism pathway |
| --- | --- | --- | --- | --- | --- |
|  |  | B/A | C/A | C/B |  |
| P12276 | FAS，fatty acid synthase | 1.40 | － | 0.84 | Synthesis of fatty acids |
| P36189 | fatty acid synthase isoform X1 | 1.37 | 1.25 | － |  |
| O00763 | ACC， acetyl-CoA carboxylase | 1.33 | 1.21 | － |  |
| KKF18473.1/  XM_010750141.1 | acetyl-CoA carboxylase 1 isoform X3 | 1.69 | 1.45 | 0.86 |  |
| Q9QUJ7 | long-chain acyl-CoA synthetase | － | 0.70 | － |  |
| Q8JJ04 | Fatty acid-binding protein （FABP） | 1.21 | 1.28 | － | Transfer of fatty acids |
| ADZ76526.1 | Apolipoprotein（14 kDa） | － | － | 0.82 |  |
| Q8VCI0 | phospholipase B-like 1 | － | 1.26 | 1.33 | Phospholipid decomposition |
| K04550 | low-density lipoprotein receptor-related protein 1 (alpha-2-macroglobulin receptor)（LRP1） | － | 1.22 | － | Lipoprotein metabolism |
| Q9BX93 | group XIIB secretory phospholipase A2（sPLA2） | 0.76 | － | － | Phospholipid synthesis |
| Q8K4F2 | Arachidonate 15-lipoxygenase B | － | － | 0.78 | Lipid synthesis |
| Q5F4A1 | carnitine O-palmitoyltransferase 1, liver isoform-like isoform X1 [Stegastes partitus] （CPT-1） | 0.82 | 0.80 | － | Oxidative decomposition of fatty acids |
| Q9UKG9 | peroxisomal carnitine O-octanoyltransferase | 0.76 | － | 1.21 |  |
| P13437 | acetyl-Coenzyme A acyltransferase (ACAC) | 0.82 | 0.81 | - |  |
| Q92038 | stearoyl-CoA desaturase 1a（SCD-1a） | － | 0.77 | － | UFA generation |

The symbol “-“ indicates that the difference multiple is between 0.83 and 1.20, this also applies for the following tables.

**Table S4** Variation of Differentially expressed proteins associated with glycometabolism

| Protein IDs | Protein name | Fold change | | | Metabolism pathway |
| --- | --- | --- | --- | --- | --- |
|  |  | B/A | C/A | C/B |  |
| O42153 | Glucose-6-phosphatase | 0.85 | 0.75 | － | gluconeogenesis |
| Q90592 | solute carrier family 2 facilitated glucose transporter member 2（GLUT2） | － | 1.25 | － | Transfer of glucose |
| P47859 | ATP-dependent 6-phosphofructokinase （ATP-PFK） | 0.79 | － | 1.24 | glycolysis |
| P26325 | alcohol dehydrogenase（ADH） | 0.67 | － | － |  |
| Q16875 | 6-phosphofructo-2-kinase/fructose-2,6-bisphosphatase 3-like isoform X1 | － | 0.79 | － |  |
| Q02974 | Ketohexokinase（KHK） | － | 1.22 | 0.85 |  |
| Q15119 | Pyruvate dehydrogenase [lipoamide] kinase isozyme 2（PDK2） | 0.76 | 0.72 | － | Linking glycolysis, TCA and ATP generation |
| Q91V92 | ATP-citrate synthase （ATP-CS） | 1.38 | 1.32 | － | TCA cycling |
| P53396 | ATP-citrate synthase, partial | 1.95 | 1.84 | － |  |
| Q04467 | isocitrate dehydrogenase [NADP], mitochondrial isoform X1 (ICDH X1) | 1.23 | － | － |  |
| Q26365 | ADP,ATP carrier protein(AACP) | 1.35 | 1.33 | － | oxidative phosphorylation |
| P07926 | mitochondrial ATP synthase lipid-binding protein | － | － | 1.22 |  |
| P24140 | UDP-N-acetylglucosamine--dolichyl-phosphate N-acetylglucosamine phosphotransferase | 1.23 | － | － |  |
| P00025 | Cytochrome c | － | － | 0.69 | respiratory metabolism |
| O13082 | cytochrome c oxidase subunit 6A | 0.86 | － | － |  |

**Table S5** Variation of Differentially expressed proteins associated with oxidative stress

| Protein IDs | Protein name | Fold change | | |
| --- | --- | --- | --- | --- |
|  |  | B/A | C/A | C/B |
| Q9DED4 | cold inducible RNA binding protein（CIRP） | 1.26 | － | － |
| O35162 | heat shock 70 kDa protein 13 | 0.83 | 0.78 | － |
| Q90593 | heat shock 70kDa protein 5 | － | 0.83 | － |
| P11884 | aldehyde dehydrogenase (NAD+) （ALDH） | － | 1.20 | － |
| P26325 | S-(hydroxymethyl)glutathione dehydrogenase / alcohol dehydrogenase | 0.67 | － | － |
| P80894 | alpha-class glutathione S-transferase | 1.29 | － | 0.83 |
| Q6NYL3 | Peroxisomal bifunctional enzyme | － | 1.24 | － |
| Q64462 | cytochrome P450 |  | 1.35 |  |

**Table S6** Variation of Differentially expressed proteins associated with immune response

| Protein IDs | Protein name | Fold change | | |
| --- | --- | --- | --- | --- |
|  |  | B/A | C/A | C/B |
| P06314 | immunoglobulin light chain precursor | 1.31 | 1.35 | － |
| P20957 | immunoglobulin delta heavy chain | － | 1.33 | － |
| AAX78206.1  AY885703.1  (Epinephelus coioides) | immunoglobulin mu heavy chain | － | 1.66 | 1.32 |
| P01771 | Ig heavy chain V-III region VH26 | － | 1.24 | － |
| Q03626 | Alpha-2-macroglobulin | 1.21 | 1.43 | － |
| Q8CFR0 | complement C1q-like protein 3 | 0.85 | 1.23 | 1.46 |
| P79755 | complement component C9 | 0.65 | － | － |
| P08649 | complement component 4 | 1.27 | 1.39 | － |
| P83270 | Hemoglobin subunit alpha-1 | 1.24 | － | － |
| P49947 | ferritin middle subunit, partial | 1.44 | － | 0.78 |
| P17213 | bactericidal permeability increasing protein/lipopolysaccharide binding protein（BPI/LBP） | － | 1.25 | 1.21 |
| Q9UG22 | GTPase IMAP family member 4（Gimap4） | 1.32 | 1.54 | － |

**Table S7** Variation of Other Differentially expressed proteins associated with protein and amino acid metabolism

| Protein IDs | Protein name | Fold change | | |
| --- | --- | --- | --- | --- |
|  |  | B/A | C/A | C/B |
| P86810 | L-amino-acid oxidase （LAAO） | 0.68 | 0.74 | － |
| O89001 | carboxypeptidase D isoform X2 | 1.26 | － | － |
| [Q6NYL5](..\\201801转录组结果\\附件\\4.GeneFunctionalAnnotation\\4.1.GeneFunctionalAnnotation\\Blast_Swissprot.xls#Blast_Swissprot!C4326) | alanine aminotransferase 1 | 1.27 | － | 0.78 |
| A7MBU6 | tryptophan 2,3-dioxygenase isoform X1 | 1.22 | － | － |
| P13437 | glutamyl aminopeptidase | 0.68 | 0.62 | － |
| O43272 | agmatinase | 1.30 | － | － |
| Q90XD2 | proline dehydrogenase | 1.26 | － | － |
| Q14117 | dihydropyrimidinase | 1.25 | － | 0.81 |
| Q5SUR0 | phosphoribosylformylglycinamidine synthase | 1.22 | － | 0.84 |
| Q6P132 | tax1-binding protein 1 homolog B-like [Larimichthys crocea] （Tax1-BP1B） | 1.31 | － | 0.83 |
| Q07157 | tight junction protein 1（TJP1） | 1.23 | 1.35 |  |
| Q14692 | ribosome biogenesis protein BMS1 homolog isoform X1 | 0.83 | － | － |
| Q99PL5 | ribosome-binding protein 1 | － | 1.43 | － |
| P09645 | tubulin alpha chain | － | － | 1.51 |
| Q7ZUC2 | calcium-binding protein(CBPs） | 0.79 | 0.76 | － |
| K03084 | phosphatidylinositol-3,4,5-trisphosphate 5-phosphatase 1 | － | － | 1.20 |
| Q1L994 | protein phosphatase 1 regulatory subunit 37（PP1，37） | 0.81 | － | － |
| Q9HBY8 | serine/threonine-protein kinase Sgk2 | 1.22 |  |  |
| P70336 | rho-associated protein kinase 2 isoform X1 [Larimichthys crocea]（ROCK） | 1.33 | － | － |
| P67998 | ribosomal protein S6 kinase beta（RBS6KB） | 1.25 | 1.33 | － |
| Q5RFJ7 | ribose-phosphate pyrophosphokinase 2-like（PRPS2） | 1.20 | 1.25 | － |
| Q6DEB4 | cAMP-regulated phosphoprotein 19-like | 1.33 | 1.32 | － |
